# Fabrication of the SiC/HfC Composite Aerogel with Ultra-Low Thermal Conductivity and Excellent Compressive Strength

**DOI:** 10.3390/gels10050292

**Published:** 2024-04-24

**Authors:** Wei Wang, Qi You, Zhanwu Wu, Sheng Cui, Weimin Shen

**Affiliations:** 1 College of Materials Science and Engineering, Nanjing Tech University, Nanjing 210009, China; pub0818wangwei@163.com (W.W.); 13813967958@163.com (Q.Y.); 2 Jiangsu Collaborative Innovation Center for Advanced Inorganic Function Composites, Nanjing Tech University, Nanjing 211800, China; 3 Shanghai Space Propulsion Technology Research Institute, Huzhou 313000, China; 18351926702@163.com (Z.W.); sunhuisd@163.com (W.S.)

**Keywords:** SiC/HfC composite aerogel, sol-gel, atmospheric pressure drying, carbothermal reduction, thermal insulation, compressive strength

## Abstract

Aerogels, as a new type of high-temperature-resistant insulation material, find extensive application in aerospace, high-temperature industrial furnaces, new energy batteries, and various other domains, yet still face some limitations such as inadequate temperature resistance and pronounced brittleness. In this work, SiC/HfC composite aerogels were prepared through a combination of sol-gel method, atmospheric pressure drying technique, and carbothermal reduction reaction. The effects of different molar ratios, calcination time, and temperatures on the microstructural features and physicochemical properties of the resulting SiC/HfC composite aerogels were investigated. The aerogel exhibited an elevated BET-specific surface area of 279.75 m^2^/g, while the sample displayed an extraordinarily low thermal conductivity of 0.052 W/(m·K). Most notably, the compressive strength reached an outstanding 5.93 MPa after a carbonization temperature of 1500 °C, far exceeding the values reported in prior aerogel studies. This research provided an innovative approach for advancing the development of carbide aerogels in the realm of high-temperature applications.

## 1. Introduction

Aerogels, characterized by a distinctive three-dimensional network structure formed from colloidal particles or polymer molecular chains, represent a class of nano-porous materials with notable properties such as high specific surface area, low thermal conductivity, ultra-low density, and significant porosity. These features render aerogels highly versatile, finding applications in thermal insulation and insulating materials [1], energy storage and conversion [2], biomedical applications [3], and catalysis [4], as well as adsorption and separation technologies [5]. Despite the remarkable thermal stability and low thermal conductivity exhibited by oxide aerogels like SiO_2_ [6], Al_2_O_3_ [7], ZrO_2_ [8], and composite oxide aerogels [9,10,11], the inherent vulnerability of SiO_2_ aerogels to structural collapse and densification at temperatures exceeding 800 °C poses a considerable challenge. This limitation hinders the widespread use of oxide aerogels in demanding high-temperature environments, a growing challenge in various industries and scientific fields.

Carbide aerogels [12], distinguished by their outstanding physico-chemical properties including ultra-high temperature resistance, low density, high porosity, low thermal conductivity, chemical stability, and high specific strength, emerge as compelling alternatives to overcome the constraints associated with SiO_2_ aerogels in high-temperature thermal protection systems. Silicon carbide (SiC) aerogel, recognized for its outstanding performance, has emerged as the pioneer and is an extensively investigated carbide aerogel. The inception of SiC aerogel can be traced back to a serendipitous discovery in 2004 when Lu et al. [13] fortuitously obtained a SiC/Silica nanocomposite through high-temperature heat treatment of a composite material comprising graphite carbon nanofibers/SiO_2_ aerogel in an inert atmosphere. Notably, during this initial phase, they had not yet offered a comprehensive definition or performance assessment of SiC aerogel. In 2010, Leventis et al. [14] pioneered the creation of a bulk porous SiC aerogel using polyacrylonitrile crosslinked silica aerogel as a precursor, laying the foundation for subsequent research on SiC aerogels. In 2014, Kong et al. [15] crafted an innovative bulk SiC aerogel from RF/SiO_2_ composite aerogel as a precursor, and delved into the metamorphosis of its morphology and pore structure during heat treatment while systematically scrutinizing the thermal insulation capability of SiC aerogels. Chabi et al. [16] acquired SiC aerogels with a density as minimal as 0.009 g/cm^3^, employing foam graphene as a carbon precursor. Owing to the intrinsic flaws of SiC aerogels, particularly their constrained strength, certain scholars have pursued the fabrication of C, SiO_2_, and SiC composite aerogels [17,18,19] through meticulous control of heat treatment temperature and duration to elevate the aerogels’ thermal and oxidative resilience. Furthermore, some researchers have augmented the capabilities of SiC composite aerogels in the harshest environments by incorporating additional constituent materials such as TiO_2_ [20], mullite [21], and Si_3_N_4_ [22] into the sol-gel synthesis process.

As a single compound with the highest documented melting point (about 3900 °C), HfC is widely used in the chemical and aerospace industries owing to its unique blend of covalent, metallic, and ionic bonds, which provides excellent properties such as high strength, high hardness, high melting point, excellent wear resistance, and high chemical stability [23,24,25,26]. In 2016, Patra et al. [27] fabricated nanoscale HfC particles at 1300 °C through the utilization of polycondensation and carbothermal reduction reactions involving pectin and hafnium tetrachloride. Additionally, Wang et al. [28] synthesized hafnium carbide nanoparticles employing waste plastics (including waste polyethylene, waste polytetrafluoroethylene, and polyvinyl chloride) as the carbon source and hafnium dioxide as the hafnium precursor. Kim et al. [29] utilized HfO_2_ as the source of hafnium, while phenolic resin served as the carbon precursor, yielding ultrafine, high-purity HfC powders via spark plasma sintering at 1600 °C for 1 h. Their results emphasized the fact that the de-agglomeration of the HfO_2_ powders before the carbothermic reduction process showed a reduction in the residual carbon content within the HfC powders. Luan et al. [30] fabricated HfC-modified C/SiC (C/SiC-HfC) composites through chemical vapor deposition (CVI) and reactive melting infiltration (RMI). The incorporation of HfC greatly enhanced the antioxidative characteristics of the materials. Tian et al. [31] used a catalyst-assisted chemical vapor deposition technique to grow HfC nanowires (HfCnw) within carbon felt, resulting in HfCnw-reinforced carbon/carbon (HfCnw-C/C) composites. The study showed that even with a small amount (1.75%) of HfCnw, the composite materials exhibited significantly improved oxidation resistance. Additionally, Bae et al. [32] dispersed HfC as a reinforcing phase within silicon carbide fiber-reinforced silicon carbide composites (SiC_f_/SiC), consequently enhancing the stress relaxation parameters and apparent activation energy of the material, thereby ameliorating its high-temperature creep behavior. In the above studies, HfC is commonly used as a reinforcing agent to increase the strength and oxidation resistance of materials. While silicon carbide aerogel has exceptional thermal resistance and insulating properties, it is hampered by significant brittleness and inadequate antioxidant properties under extreme conditions. This work proposes an innovative approach to address these shortcomings by synthesizing SiC/HfC composite aerogels via in situ generation of HfC within SiC aerogel. This method significantly improves the thermal resilience and compressive strength of the materials, thereby meeting the stringent requirements for thermal protection in extreme environments.

The sol-gel technique [33], a key step in aerogel preparation process, involves dissolving raw materials such as metal–alcohol salts in water or alcohol and then converting them into gels by hydrolysis/alcoholysis heating and stirring. Widely used in various fields of nanomaterial synthesis, it was characterized by precise control of the chemical components, ensuring thorough and uniform mixing, with additional benefits including low reaction temperatures, cost effectiveness, and high purity, together with uniform particle size distribution in the products. In aerospace, Passaro et al. [34] have used sol-gel technology to produce hydrophobic cannabis particles and incorporated them into epoxy resin coatings to enhance the hydrophobicity, anti-icing properties, and thermal stability of aerospace substrates. Similarly, in the field of ultra-high temperature ceramics [35], the ability of the sol-gel method to regulate the internal pore structure is finding wide application in ultra-high temperature porous ceramics, facilitating the creation of materials that are resistant to high temperatures, sinterable, and lightweight. Singh et al. [36] achieved LaFeO_3_-modified bismuth sodium titanate ceramics via sol-gel methodology, exploiting their excellent dielectric, optical, magnetic, piezoelectric, and catalytic properties, which are widely used in memory, displays, fuel cells, and capacitors. The homogeneous mixing of reactants during sol-gel processes promotes a uniform nanoporous structure within aerogels, which improves the overall thermal insulation performance, making sol-gel the predominant method for aerogel preparation [37].

In this work, we have prepared a composite wet gel by blending HfO_2_ sol, SiO_2_ sol, and RF mixed solution using the sol-gel technique [33], which was then dried using cost-effective atmospheric pressure drying technology to yield the precursor aerogel. The sol-gel process, facilitated by catalysts like HCl, a hydrolysis product of the hafnium source, drives irreversible chemical reactions to produce final samples. Furthermore, during the aging process of the sample, the introduction of n-hexane, characterized by lower surface tension, effectively addresses the issue of aerogel cracking and fracture induced by surface tension during normal pressure drying. Ultimately, building upon the advancements made by predecessors in the realm of sol-gel synthesis of porous ultra-high temperature ceramics [35], the SiC/HfC composite aerogels were synthesized through a carbothermal reduction process conducted under an inert atmosphere of argon or helium. The effects of varying molar ratios, heat treatment temperatures, and durations on the microstructure and properties of the aerogels were investigated, including thermal resistance, compressive strength, and oxidation resistance of SiC/HfC composite aerogels.

## 2. Results and Discussion

### 2.1. Synthetic Route of SiC/HfC Composite Aerogel

**Figure 1:**
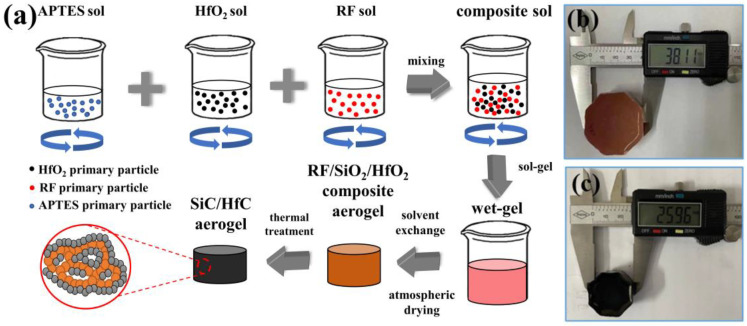
(**a**) Preparation process diagram of SiC/HfC composite aerogel, the macro sample diagram of (**b**) RF/SiO_2_/HfO_2_ composite aerogel, and (**c**) SiC/HfC composite aerogel.

Figure 1 shows the process flowchart for the preparation of SiC/HfC composite aerogel, along with the macroscopic representation of the composite aerogel before and after carbothermal reduction. As shown in the figure, HfOCl_2_·8H_2_O is dissolved in deionized water, while APTES and RF are dissolved in ethanol and carefully mixed. The solutions are then mixed and stirred to produce the RF/SiO_2_/HfO_2_ composite sol. During the sol-gel reaction process, HfOCl_2_ undergoes hydrolysis to form HCl, resulting in an acidic solution environment. Under excess formaldehyde conditions (with a fixed R/F molar ratio of 1:2), multiple electrophilic substitution reactions occur between formaldehyde and the phenyl rings of resorcinol within the acidic milieu. This leads to the formation of various intermediate products containing multiple hydroxymethyl groups attached to the phenyl rings of resorcinol. These intermediate products subsequently undergo condensation with each other and with resorcinol and formaldehyde, forming a continuous network structure. Furthermore, surface hydroxyl groups on hafnium oxide and alumina (hydrolysis products of the hafnium and silicon sources, respectively) undergo a series of hydrolysis–condensation reactions with the hydroxymethylated intermediates of resorcinol, resulting in the formation of RF/SiO_2_/HfO_2_ composite sol molecules containing Hf-O-Hf and Si-O-Si bonds, as well as Hf-O-C and Si-O-C bonds. These composite sol molecules eventually undergo further reactions in the oven at a certain temperature to form RF/SiO_2_/HfO_2_ composite wet gels, which is then subjected to normal pressure drying to obtain an RF/SiO_2_/HfO_2_ composite aerogel, as shown in Figure 1b. Finally, the SiC/HfC composite aerogel is synthesized by high temperature carbonization and carbothermal reduction reaction (as shown in Figure 1c), exhibiting an internal double network structure.

### 2.2. Chemical Composition and Structural Analysis

**Figure 2:**
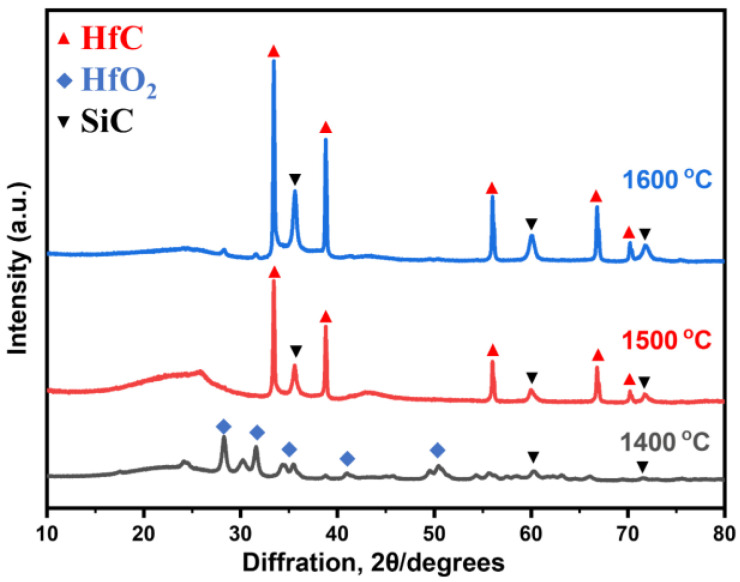
XRD analysis of SiC/HfC composite aerogel after different carbon thermal reduction temperatures.

Figure 2 shows the XRD pattern of the specimen after heat treatment of the RF/SiO_2_/HfO_2_ composite aerogel at different temperatures. The figure indicates that the phases of the SiC/HfC composite aerogel undergo changes with increasing heat treatment temperature. Notably, obvious peaks appeared at 24.64°, 28.30°, 31.76°, and 50.46° when heat-treated at 1400 °C, correspond to the (0 1 1), (−1 1 1), (1 1 1), and (0 2 2) planes of t-HfO_2_, respectively (JCPDS: No. 53-0550). Concurrently, the diffraction peaks attributed to the SiC phase manifest at 35.66°, 60.15°, and 71.83° (JCPDS: No. 29-1129) [14], with the peak at 35.66° agreeing well with the diffraction peak of HfO_2_. As the temperature rises to 1500 °C, HfO_2_ is transformed into HfC via the carbothermal reduction process, the peaks observed at 33.4°, 38.8°, 56.1°, 66.8°, and 70.1° are attributed to the (1 1 1), (2 0 0), (2 2 0), (3 1 1). and (2 2 2) planes of the HfC phase, respectively (JCPDS: No. 65-0964) [26], while the intensity of the peaks of SiC and HfC increase significantly with increasing temperature up to 1600 °C.

**Figure 3:**
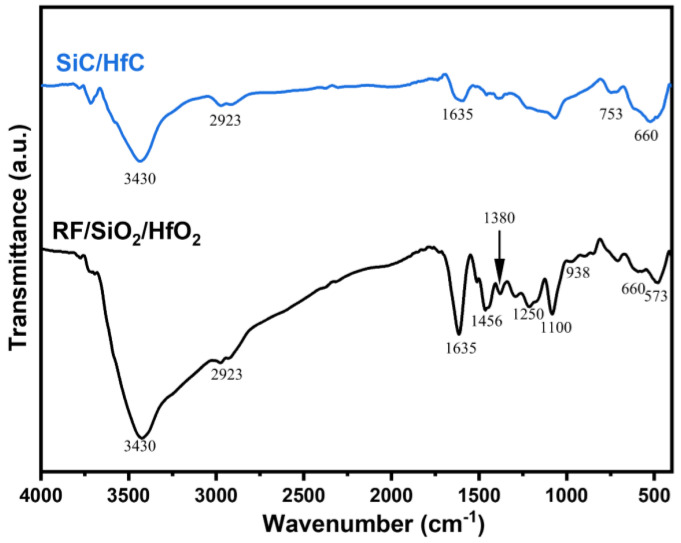
FT-IR plots of RF/SiO_2_/HfO_2_ composite aerogel and SiC/HfC composite aerogel.

The FT-IR spectra of RF/SiO_2_/HfO_2_ composite aerogel and SiC/HfC composite aerogel are depicted in Figure 3. There is an obvious broad absorption band at 3430 cm^−1^, which is attributed to water vapor adsorption on the prepared samples, inducing the antisymmetric stretching vibration of hydrogen bonds between -OH bonds. Similarly, the absorption band at 1635 cm^−1^ arises from water molecules adsorbed within the aerogel. Additionally, the absorption bands at 2923 cm^−1^, 1456 cm^−1^, and 1380 cm^−1^ correspond to -CH_2_ absorption within the resorcinol–formaldehyde system, C-C in the benzene ring skeleton [38], and C-H stretching vibration in -CH_2_, respectively. The band at 1250 cm^−1^ is attributed to bending vibration within the -OH group of resorcinol. Furthermore, the absorption band at 938 cm^−1^ corresponds to the C-O bond in Hf-O-C. Additionally, absorption bands at 753 cm^−1^, 660 cm^−1^, and 573 cm^−1^ correspond to the Hf-O bond [39], while the absorption band at 1100 cm^−1^ corresponds to the stretching vibrational absorption peak in Si-O-Si [23].

**Figure 4:**
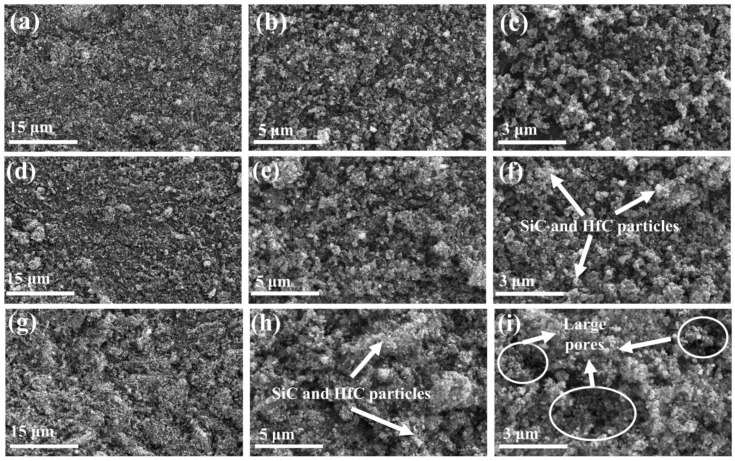
SEM images of SiC/HfC composite aerogel at different heat treatment temperatures: (**a**–**c**) 1400 °C, (**d**–**f**)1500 °C and (**g**–**i**) 1600 °C.

Figure 4 shows SEM images of SiC/HfC composite aerogels at different heat treatment temperatures. The images demonstrate that the SiC/HfC composite aerogels are composed of stacked nanoparticles, forming a nanoporous network structure. The aerogels exhibit a disordered mesoporous structure, which is primarily attributed to the presence of three distinct components in the sol-gel process, namely, SiO_2_, HfO_2_, and RF, and the differential crosslinking rates of these components. At a carbon thermal reduction temperature of 1400 °C (as shown in Figure 4a–c), the internal particle distribution within the aerogel appears to be relatively uniform. It mainly consists of small particles such as HfO_2_, SiO_2_, and carbon particles with sizes ranging from 20–50 nm, while XRD results indicate a minor presence of SiC. When the heat treatment temperature is increased to 1500 °C, HfO_2_ particles with a smaller size are observed. However, SiO_2_ and the carbon aerogel undergo carbothermal reduction, resulting in larger HfC and SiC particles. Additionally, Figure 4e shows a decrease in internal pore size, a denser network structure, and the emergence of a few macropores. Upon further escalation of the heat treatment temperature to 1600 °C, the prevalence of macropores increases, as evidenced in Figure 4g–i. As a result, the carbon aerogel scaffold is depleted, causing accumulation and agglomeration of the generated HfC and SiC particles, resulting in a tighter and denser internal network structure. Furthermore, extended exposure to high-temperature heat treatment will lead to a partial collapse of the internal pore structure of the aerogel, resulting in the formation of supplementary macroporous architecture.

**Figure 5:**
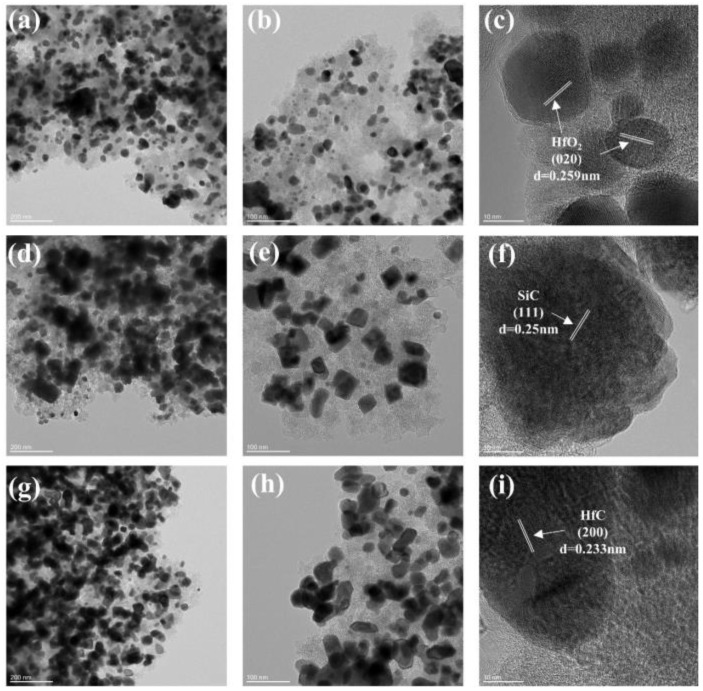
TEM images of SiC/HfC composite aerogel at different heat treatment temperatures: (**a**–**c**) 1400 °C, (**d**–**f**) 1500 °C and (**g**–**i**) 1600 °C.

Figure 5 shows TEM images of SiC/HfC composite aerogels after different heat treatment temperatures. It is worth noting that for the samples treated at 1400 °C, Figure 5a–c indicates the presence of HfO_2_ and SiO_2_ particles within the composite aerogel, with a particle size distribution ranging from 10 to 30 nm, while the particles are located in the disordered realm of the amorphous carbon aerogel. Larger particles, with diameters exceeding 50 nm, are present as intermediates of the carbothermal reduction process of HfO_2_, identified as HfC_x_O_y_. In addition, lattice stripes corresponding to the (0 2 0) crystal facets of HfO_2_, with a crystal plane spacing of 0.259 nm, are visible in Figure 5c. As the heat treatment temperature increases, large particles begin to move closer to each other within the sample, as shown in Figure 5d–i, which indicates the ongoing carbothermal reduction process of oxide particles. At the same time, SiC and HfC emerge abundantly as the carbon aerogel depletes, which is consistent with the XRD findings. Additionally, the lattice fringes of SiC exhibit a crystal plane spacing of 0.25 nm [40], which is representative of the (1 1 1) crystal plane. On the other hand, lattice fringes with a crystal plane spacing of 0.233 nm correspond to the (2 0 0) crystal plane of HfC.

**Figure 6:**
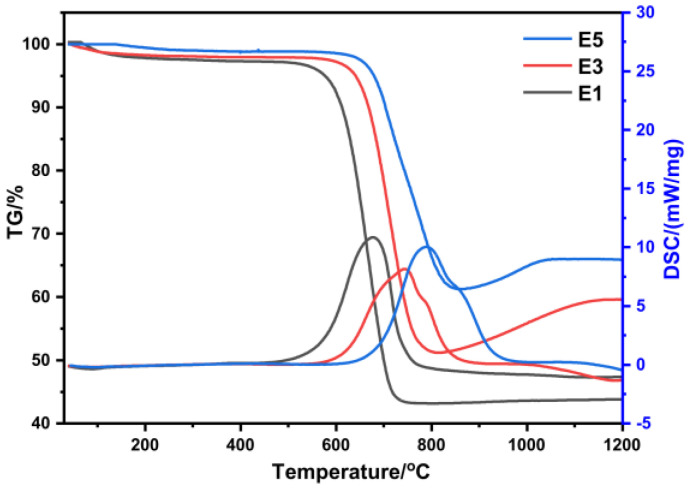
TG–DSC curves of SiC/HfC aerogels under flowing air during heat treatments.

The antioxidant efficacy of SiC/HfC composite aerogels was assessed by subjecting samples with varying Si/Hf molar ratios to TG–DSC analysis in an air environment, as shown in Figure 6. In the first stage temperature range (30–600 °C), a mass loss rate of 2–4% was observed, primarily due to the evaporation of physically adsorbed water molecules and the decomposition of residual organic functional groups within the samples. In the second phase, occurring at approximately 600–800 °C, characterized by a mass reduction of up to 57%. This phenomenon arises from the liberation of CO and CO_2_ gases consequent to the oxidation of residual amorphous carbon within the specimen [23], resulting in a substantial decrease in mass. Furthermore, an elevation in the Si/Hf molar ratio correlates with a reduction in sample mass loss, accompanied by a rightward shift in the heat absorption peak. This shift signifies that the inclusion of silicon source adeptly enhances the material’s degree of carbothermal reduction and resistance to oxidation. Subsequently, during the third stage, the sample mass experiences an increment primarily attributed to the oxidation of SiC and HfC. Notably, the mass of the E5 specimen remains unaltered even after reaching 1000 °C, predominantly owing to the formation of a molten SiO_2_ layer on the aerogel’s surface, which efficaciously impedes the diffusion of oxygen molecules.

### 2.3. Thermogravimetric and Pore Morphology Analysis

**Figure 7:**
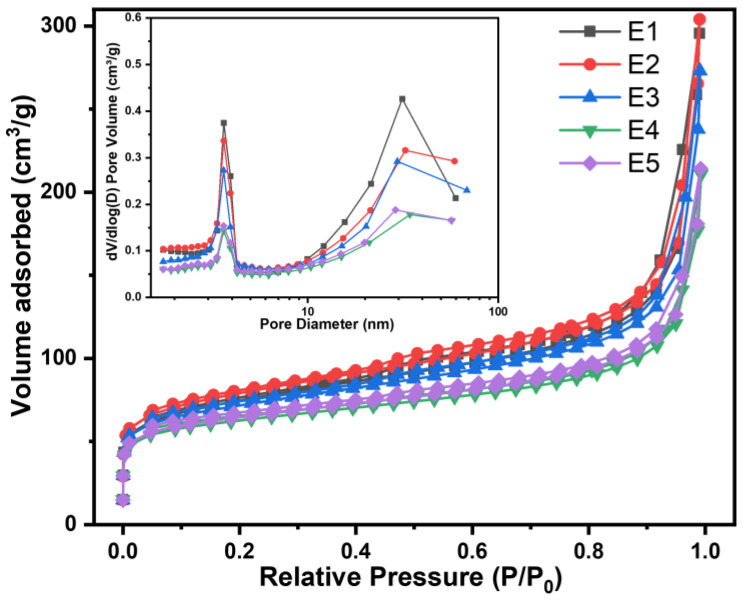
N_2_ adsorption/desorption curves and pore size distribution curves of SiC/HfC composite aerogels with different Si/Hf molar ratios.

Figure 7 shows the N_2_ adsorption/desorption curves and pore size distributions of SiC/HfC composite aerogels with varying Si/Hf molar ratios. The adsorption–desorption isotherms of SiC/HfC composite aerogels conform to type IV classification per the IUPAC standards, indicating a mesoporous nature with pore dimensions ranging from 2 to 50 nm [41]. The H1-type hysteresis loop is also displayed. At a relative pressure (P/P0) below 0.05, the curve deviates from the *Y*-axis, indicating the presence of micropores with strong adsorption affinity within the aerogel matrix. Adsorption reaches equilibrium at a relative pressure (P/P0) of 0.5, which is attributed to multilayer adsorption occurring on the exterior surface of meso- and macropores. Conversely, when the relative pressure (P/P0) is close to 1, no adsorption plateau is observed. This suggests that while the aerogel is predominantly mesoporous, there is a segment characterized by a macroporous structure, which is consistent with SEM analysis findings. Table 1 provides specific details on the aerogel specimens’ specific surface area, average pore size, and pore volume. The specific surface area of the SiC/HfC composite aerogel material exhibited a pattern of increase followed by decrease with the elevation of the Si/Hf molar ratio, peaking at 279.75 m^2^/g for the sample with a Si/Hf molar ratio of 0.25:1. Moreover, analysis of the average pore diameter revealed that the SiC/HfC composite aerogel manifested as a quintessential mesoporous substance, boasting an average pore diameter of approximately 12 nm. Concurrently, the pore volume of the composite aerogel shows a general decrease with increasing Si/Hf molar ratios.

### 2.4. Compressive Strength and Thermal Conductivity Analysis

Table 2 presents the density, compressive strength, and thermal conductivity of SiC/HfC composite aerogels. It is worth noting that the bulk density of the aerogel increases with the Si/Hf molar ratio, while the apparent density initially increases and subsequently decreases. The bulk density of the SiC/HfC composite aerogel is only 0.35 g/cm^3^, while its compressive strength is 5.93 MPa, which is a significant improvement over the previously published works [12,17,18,19,42]. Moreover, the SiC/HfC composite aerogel exhibits an exceptional thermal conductivity of 0.052 W/(m·K) at room temperature, consistent with the findings from the BET analysis.

## 3. Conclusions

In this investigation, we have synthesized a novel variety of SiC/HfC composite aerogel, showcasing exceptional physical and chemical attributes through the sol-gel method and carbothermal reduction reaction. Notably, the sample presents a remarkably large specific surface area of 279.75 m^2^/g, indicating its abundant porous structure. In addition, the SiC/HfC composite aerogel displays an exceptionally low thermal conductivity of 0.053 W/(m·K). Furthermore, while retaining exceptional thermal insulation properties, the SiC/HfC composite aerogel achieved an impressive compressive strength of 5.93 MPa. The remarkable compressive strength of the SiC/HfC composite aerogel, exceeding that of previously published works, positions it as an ideal choice for ultra-high temperature and harsh application environments. This new lightweight insulation material, combining excellent thermal insulation performance with exceptional strength, is particularly well-suited for areas requiring high-temperature insulation, particularly in the increasingly demanding aerospace industry.

## 4. Materials and Methods

### 4.1. Materials

HfOCl_2_·8H_2_O and APTES were purchased from Alfa Aesar Co., Ltd., Shanghai, China. Resorcinol was purchased from Xilong Chemical Co., Ltd., Chengdu, China. Formaldehyde was purchased from Shanghai Lingfeng Chemical Reagent Co., Ltd., Shanghai, China. Absolute ethyl alcohol and deionized water were provided by Wuxi City Yasheng Chemical Co., Ltd., Wuxi, China, and Nanjing Wanqing chemical Glass ware & Instrument Co., Ltd., Nanjing, China., respectively.

### 4.2. Synthesis

This investigation utilized HfOCl_2_·8H_2_O as the hafnium source, APTES as the silicon source, and RF as the carbon source. Ethanol was used as the solvent, and deionized water was used as the hydrolysate. No reactants or solvents were purified before use, and no catalysts were introduced during the experiments.

The aerogel preparation followed this methodical procedure: firstly, HfOCl_2_·8H_2_O was added to a beaker, followed by deionized water. The synthesis of the HfO_2_ composite sol and SiO_2_ sol involved several steps. The mixture was stirred magnetically at 50 °C for 60 min until complete hydrolysis occurred. In parallel, APTES was dissolved in a mixed solvent of ethanol and water to produce the SiO_2_ sol. In a separate container, resorcinol (R) and formaldehyde (F) were blended with anhydrous ethanol to yield the RF sol after 30 min of magnetic stirring. The RF sol was then combined with the HfO_2_ sol, while the SiO_2_ sol was mixed with an appropriate amount of ammonia. This resulted in the formation of the RF/SiO_2_/HfO_2_ composite sol. The composite sol was then poured into a plastic mold and left in a dry oven at 60 °C for 30–150 min to fabricate the RF/SiO_2_/HfO_2_ composite wet gel. After the composite wet gel was prepared, solvent replacement was carried out using anhydrous ethanol to eliminate any residual unreacted solvent. The process of replacement was repeated every 12 h for 6–8 cycles. After that, hexane was used for another round of solvent replacement, with replacements carried out every 8 h for 6–9 cycles. Finally, the composite wet gel was dried under atmospheric pressure in a high-temperature drying oven. The temperature was gradually increased to 50 °C, 60 °C, and 70 °C for 30 min, resulting in the production of RF/SiO_2_/HfO_2_ composite aerogel. The SiC/HfC composite aerogel was prepared by subjecting the RF/SiO_2_/HfO_2_ composite aerogel to high-temperature carbonization at 800 °C for 3 h in a tube furnace under an inert helium gas environment, resulting in the C/SiO_2_/HfO_2_ composite aerogel. The final product, bulk SiC/HfC composite aerogel, was obtained through carbothermal reduction treatment at temperatures of 1400 °C, 1500 °C, and 1600 °C. Of these, the samples are designated E1, E2, E3, E4, and E5, with the corresponding Si/Hf molar ratios being 0.1:1, 0.25:1, 0.5:1, 1:1, and 1.5:1 respectively.

The high-temperature heat treatment was performed using a GSL-1750-KS single-temperature-zone atmosphere tube furnace, manufactured by Hefei Techchip Materials Co. (Hefei, China). The furnace is capable of reaching temperatures up to 1750 °C and was meticulously calibrated for precise temperature control. The heat treatment process employed a gradual ramp-up program, with the temperature increasing at a rate of 2 °C per minute. The process was conducted in a helium (He) atmosphere with a continuous airflow of 100–150 mL/min throughout.

### 4.3. Characterizations

Thermal gravimetric analysis (TGA) and differential scanning calorimetry (DSC) were conducted using a NETZSCH STA449 F3 thermogravimetric analyzer with a heating rate of 10 °C/min. X-ray diffraction (XRD) data were obtained using a Rigaku Smartlab equipped with a 9 KW X-ray source and CuKα1 radiation (λ = 0.15406 nm). Scanning electron microscopy (SEM) was carried out with a Phenom Pharos G2 field-emission scanning electron microscope. Surface area, pore distribution, and pore volume measurements were conducted using a Micromeritics ASAP 2460 instrument. Infrared spectral analysis was performed using a Thermo Scientific Nicolet iS20 infrared spectrometer. Transmission electron microscopy (TEM) imaging was carried out with an FEI Talos F200X G2 operating at 200 kV. Compressive strength testing was performed using an INSTRON 3382 testing device on samples in the form of 30 mm cubic cubes. The testing was conducted at a speed of 2.0 mm/min and at a temperature of 25 °C. To measure the thermal conductivity, carbon-fiber-mat-reinforced aerogel composites (40 mm × 40 mm × 10 mm) were degassed in a vacuum at 90 °C for 6 h and analyzed using a Hot Disk-2500 thermal constant analyzer.

## Figures and Tables

**Table 1:** Pore structures of SiC/HfC composite aerogels with various Si/Hf molar ratios.

Sample	Si/Hf Molar Ratio	BET Surface Areas (m^2^/g)	Average Pore Diameters (nm)
E1	0.1:1	265.11	11.64
E2	0.25:1	279.75	11.80
E3	0.5:1	256.61	12.32
E4	1:1	225.87	12.69
E5	1.5:1	234.06	12.02

**Table 2:** The density, compressive strength, and thermal conductivity of SiC/HfC composite aerogel with various Si/Hf molar ratios.

Sample	Bulk Density (g/cm^3^)	Compressive Strength (MPa)	Thermal Conductivity (W/(m·K))
E1	0.38	4.11	0.054
E2	0.40	5.93	0.052
E3	0.36	5.43	0.057
E4	0.35	4.57	0.060
E5	0.35	4.23	0.064

## Data Availability

The raw/processed data required to reproduce these findings cannot be shared at this time as the data also form part of an ongoing study.

## References

[1] Zhou J., Hsieh Y.L. (2020). Nanocellulose aerogel-based porous coaxial fibers for thermal insulation. Nano Energy.

[2] Chhetri K., Subedi S., Muthurasu A., Ko T.H., Dahal B., Kim H.Y. (2022). A review on nanofiber reinforced aerogels for energy storage and conversion applications. J. Energy Storage.

[3] Ferreira-Gonçalves T., Iglesias-Mejuto A., Linhares T., Coelho J.M., Vieira P., Faísca P., Reis C.P. (2022). Biological thermal performance of organic and inorganic aerogels as patches for photothermal therapy. Gels.

[4] Xiong Z.C., Zhu Y.J., Wang Z.Y., Chen Y.Q., Yu H.P. (2022). Tree-inspired ultralong hydroxyapatite nanowires-based multifunctional aerogel with vertically aligned channels for continuous flow catalysis, water disinfection, and solar energy-driven water purification. Adv. Funct. Mater..

[5] Jiang J., Zhang Q., Zhan X., Chen F. (2019). A multifunctional gelatin-based aerogel with superior pollutants adsorption, oil/water separation and photocatalytic properties. Chem. Eng. J..

[6] Liu Z.H., Ding Y.D., Wang F., Deng Z.P. (2016). Thermal insulation material based on SiO_2_ aerogel. Constr. Build. Mater..

[7] Janosovits U., Ziegler G., Scharf U., Wokaun A. (1997). Structural characterization of intermediate species during synthesis of Al_2_O_3_ aerogels. J. Non-Cryst. Solids.

[8] Liu B., Liu X., Zhao X., Fan H., Zhang J., Yi X., Gao M., Zhu L., Wang X. (2019). High-strength, thermal-stable ZrO_2_ aerogel from polyacetylacetonatozirconium. Chem. Phys. Lett..

[9] Yu H., Tong Z., Yue S., Li X., Su D., Ji H. (2021). Effect of SiO_2_ deposition on thermal stability of Al_2_O_3_-SiO_2_ aerogel. J. Eur. Ceram. Soc..

[10] Gao B., Cao J., Yao C., Mao L. (2022). High thermally insulating and lightweight Cr_2_O_3_-Al_2_O_3_ aerogel with rapid-cooling property. Appl. Surf. Sci..

[11] Yu H., Tong Z., Qiao Y., Yang Z., Yue S., Li X., Ji H. (2020). High thermal stability of SiO_2_-ZrO_2_ aerogels using solvent-thermal aging. J. Solid State Chem..

[12] An Z., Zhang R., Fang D. (2019). Synthesis of monolithic SiC aerogels with high mechanical strength and low thermal conductivity. Ceram. Int..

[13] Lu W., Steigerwalt E.S., Moore J.T., Sullivan L.M., Collins E.W., Lukehart C.M. (2004). Carbothermal transformation of a graphitic carbon nanofiber/silica aerogel composite to a SiC/silica nanocomposite. J. Nanosci. Nanotechnol..

[14] Leventis N., Sadekar A., Chandrasekaran N., Sotiriou-Leventis C. (2010). Click synthesis of monolithic silicon carbide aerogels from polyacrylonitrile-coated 3D silica networks. Chem. Mat..

[15] Kong Y., Zhong Y., Shen X., Cui S., Fan M. (2014). Effect of silica sources on nanostructures of resorcinol–formaldehyde/silica and carbon/silicon carbide composite aerogels. Micropor. Mesopor. Mat..

[16] Chabi S., Rocha V.G., Garcia-Tunon E., Ferraro C., Saiz E., Xia Y., Zhu Y. (2015). Ultralight, strong, three-dimensional sic structures. ACS Nano.

[17] Chithra A., Rajeev R., Prabhakaran K. (2022). C/SiO_2_ and C/SiC composite foam monoliths from rice husk for thermal insulation and EMI shielding. Carbon. Lett..

[18] Wu X., Shao G., Shen X., Cui S., Chen X. (2017). Evolution of the novel C/SiO_2_/SiC ternary aerogel with high specific surface area and improved oxidation resistance. Chem. Eng. J..

[19] An Z., Ye C., Zhang R., Qu Q. (2019). Multifunctional C/SiO_2_/SiC-based aerogels and composites for thermal insulators and electromagnetic interference shielding. J. Sol-Gel Sci. Technol..

[20] Chu P., Liu H., Li Y., Zhang H., Li J. (2016). Synthesis of SiC-TiO_2_ hybrid aerogel via supercritical drying combined PDCs route. Ceram. Int..

[21] Xie M., Wu X., Liu J., Zhang K. (2017). In-situ synthesis and textural evolution of the novel carbonaceous SiC/mullite aerogel via polymer-derived ceramics route. Ceram. Int..

[22] Dai D., Lan X., Wu L., Wang Z. (2022). Designed fabrication of lightweight SiC/Si_3_N_4_ aerogels for enhanced electromagnetic wave absorption and thermal insulation. J. Alloys Comp..

[23] Bargeron C.B., Benson R.C., Jette A.N., Phillips T.E. (1993). Oxidation of hafnium carbide in the temperature range 1400 to 2060 C. J. Am. Ceram. Soc..

[24] Zeng Q., Peng J., Oganov A.R., Zhu Q., Xie C., Zhang X., Dong D., Zhang L., Cheng L. (2013). Prediction of stable hafnium carbides: Stoichiometries, mechanical properties, and electronic structure. Phys. Rev. B.

[25] Kim D., Han J., Park C., Lee H.G., Park J.Y., Kim W.J. (2019). Chemical vapor deposition of dense hafnium carbide from HfCl_4_-C_3_H_6_-H_2_ system for the protection of carbon fibers. Adv. Eng. Mater..

[26] Liang H., Fang L., Guan S., Peng F., Zhang Z., Chen H., Lu C. (2020). Insights into the bond behavior and mechanical properties of hafnium carbide under high pressure and high temperature. Inorg. Chem..

[27] Patra N., Al Nasiri N., Jayaseelan D.D., Lee W.E. (2016). Low-temperature solution synthesis of nanosized hafnium carbide using pectin. Ceram. Int..

[28] Wang L., Dai W., Zhao D., Zhang F., Zhang K., Cheng Q., Qian Y. (2019). A simple route for the direct conversion of waste plastic to hafnium carbide nanoparticles at low temperature. Nanosci. Nanotech. Lett..

[29] Kim J., Lee S.J., Feng L., Silvestroni L., Sciti D., Lee S. (2020). Effect of residual excess carbon on the densification of ultra-fine HfC powder. J. Eur. Ceram. Soc..

[30] Luan X., Liu G., Tian M., Chen Z., Cheng L. (2021). Damage behavior of atomic oxygen on a hafnium carbide-modified C/SiC composite. Compos. Part B Eng..

[31] Tian S., Zhou L., Liang Z., Yang Y., Wang Y., Qiang X., Zhang Y. (2020). 2.5 D carbon/carbon composites modified by in situ grown hafnium carbide nanowires for enhanced electromagnetic shielding properties and oxidation resistance. Carbon.

[32] Bae S.G., Oh M., Lee Y., Kim S., Jeong Y.G., Lee J.M., Shin D.G. (2022). Fabrication and high-temperature performance evaluation of silicon carbide-hafnium carbide nanocomposite fiber. Ceram. Int..

[33] Bokov D., Turki Jalil A., Chupradit S., Suksatan W., Javed Ansari M., Shewael I.H., Kianfar E. (2021). Nanomaterial by sol-gel method: Synthesis and application. Adv. Mater. Sci. Eng..

[34] Passaro J., Bifulco A., Calabrese E., Imparato C., Raimondo M., Pantani R., Guadagno L. (2023). Hybrid hemp particles as functional fillers for the manufacturing of hydrophobic and anti-icing epoxy composite coatings. ACS Omega.

[35] Li F., Huang X., Liu J.X., Zhang G.J. (2020). Sol-gel derived porous ultra-high temperature ceramics. J. Adv. Ceram..

[36] Singh S., Kaur A., Kaur P., Singh L. (2023). Unveiling the high-temperature dielectric relaxation and conduction mechanisms in sol-gel derived LaFeO_3_ modified sodium bismuth titanate ceramics. J. Alloys Comp..

[37] Cai H., Jiang Y., Feng J., Zhang S., Peng F., Xiao Y., Feng J. (2020). Preparation of silica aerogels with high temperature resistance and low thermal conductivity by monodispersed silica sol. Mater. Design..

[38] Wang X., Zhou Z., Guo X., He Q., Hao C., Ge C. (2016). Ultrasonic-assisted synthesis of sodium lignosulfonate-grafted poly (acrylic acid-co-poly (vinyl pyrrolidone)) hydrogel for drug delivery. RSC Adv..

[39] Wang X., Zhang L., Wang Y. (2022). Preparation of HfC-SiC ultra-high-temperature ceramics by the copolycondensation of HfC and SiC precursors. J. Mater. Sci..

[40] Kong Y., Shen X., Cui S., Fan M. (2014). Preparation of monolith SiC aerogel with high surface area and large pore volume and the structural evolution during the preparation. Ceram. Int..

[41] Tangestaninejad S., Moghadam M., Mirkhani V., Baltork I.M., Ghani K. (2009). Alkene epoxidation catalyzed by molybdenum supported on functionalized MCM-41 containing N–S chelating Schiff base ligand. Catal. Commun..

[42] Ye L., Qiu W., Li H., Zhao A., Cai T., Zhao T. (2013). Preparation and characterization of ZrCO/C composite aerogels. J. Sol-Gel Sci. Technol..

